# Reshaping the hierarchical medical system for rare diseases: a two-tier structure and one-stop referral network

**DOI:** 10.7189/jogh.15.03005

**Published:** 2025-04-11

**Authors:** Chaoyu Lei, Ying Zuo, Fanyi Kong, Hao Chen, Haoxuan Cheng, Xuefei Song, Lufa Zhang, Huifang Zhou

**Affiliations:** 1 Department of Ophthalmology, Shanghai Ninth People's Hospital, Shanghai Jiao Tong University School of Medicine, Shanghai Key Laboratory of Orbital Diseases and Ocular Oncology, and State Key Laboratory of Eye Health, China; 2 School of International and Public Affairs, Shanghai Jiao Tong University, Shanghai, China; 3 Institute for Hospital Management, Tsinghua University, Shenzhen, Guangdong, China; 4 School of Healthcare Management, Tsinghua University Tsinghua Medicine, Beijing, China; 5 Shanghai Jiao Tong University School of Medicine, Shanghai, China; 6 Institute for Urban Governance, Shanghai Jiao Tong University, Shanghai, China; 7 Institute of Healthy Yangtze River Delta, Shanghai Jiao Tong University, Shanghai, China

Over 7000 rare diseases have been identified worldwide, affecting approximately 6–8% of the global population. Patients with rare diseases often endure lengthy diagnostic journeys, consulting multiple physicians over several years before receiving an accurate diagnosis. These challenges stem from significant shortcomings in the current hierarchical medical system, which lacks an effective framework for rare diseases and exhibits disparities in diagnostic and treatment capabilities across medical institutions. Most first- and second-level hospitals cannot diagnose and treat rare diseases, and the existing three-tier hospital grading system leads to ineffective referrals and delayed treatment. Moreover, the absence of an institutionalised system with integrated processes results in difficulties with initial diagnoses at the grassroots level, inefficient step-by-step referrals, and challenges in implementing reverse referrals. To address these issues, we propose reshaping the hierarchical medical system by classifying diagnostic and treatment capabilities into a two-tier structure, establishing an efficient one-stop referral network, building a digital platform, integrating with the existing health care system, and incentivising stakeholder engagement. This approach aims to facilitate patient-centred, comprehensive care, improve accessibility, and alleviate global health inequalities associated with rare diseases.

Over 7000 rare diseases have been identified worldwide, affecting approximately 6–8% of the global population [1]. On average, patients consult eight doctors before receiving a diagnosis, with diagnostic journeys lasting 5–8 years and some extending beyond a decade [1–3]. For instance, one study reported that 25% of patients with rare diseases experience diagnostic delays exceeding ten years, and 40% receive at least one misdiagnosis [3]. These challenges highlight the absence of an effective hierarchical medical system for rare diseases and reveal disparities in capabilities across medical institutions. First, most first- and second-level hospitals lack the ability to diagnose and treat rare diseases, while the existing three-tier hospital grading system increases the risk of ineffective referrals and delayed treatment. Second, an institutionalised system with integrated processes has not been established, resulting in difficulties with initial diagnoses at the grassroots level, inefficient step-by-step referrals, and challenges in implementing reverse referrals [4]. Lastly, the lack of an open and transparent digital network leads to significant information silos and time inefficiencies [5].

Therefore, it is crucial to reshape the hierarchical medical system and integrate digital technology for rare diseases. This approach will facilitate patient-centred, comprehensive care, improve accessibility, and alleviate global health inequalities. Simultaneously, it will enable more efficient management of medical services and reduce the burden on the health care system, contributing to the achievement of Sustainable Development Goal 3: ‘Good Health and Well-Being’.

## RESHAPING HIERARCHICAL STRUCTURE: FOLLOWING DIAGNOSTIC AND TREATMENT CAPABILITIES

A specialised classification system for medical institutions handling rare diseases should be developed by forming an expert group focussed on this area. Existing hospitals would be categorised into a two-tier system based on their ability to diagnose and treat rare diseases [6]. To define the two-tier structure, ‘first-level’ institutions are identified as those capable of providing basic services, including preliminary screening, early identification, and rehabilitation management for rare diseases. However, they lack specialised diagnostic equipment or multidisciplinary treatment teams. In contrast, ‘second-level’ institutions are designated as those with advanced diagnostic technologies, multidisciplinary teams, and the capacity to manage complex rare disease cases. These institutions should meet benchmarks such as having genetic testing facilities, biobanks, and clinical trial capabilities. Additionally, a dynamic performance evaluation mechanism should be established for medical institutions at different levels to ensure timely and effective supervision and management.

## IMPROVING THE REFERRAL PROCESS: ESTABLISHING A ONE-STOP SERVICE NETWORK

Promoting a seamless flow of care – encompassing initial screening, diagnosis, treatment, and rehabilitation – within the two-tier hierarchical structure is vital to establishing an efficient one-stop referral network. Specifically, this mechanism involves the following steps:

1. Establishing dedicated referral coordinators at ‘first-level’ institutions to guide patients through the process.
2. Creating standardised referral protocols: if ‘first-level’ institutions identify suspected rare disease cases, they can directly refer them to ‘second-level’ institutions through a digital system.
3. Referral coordinators at ‘second-level’ institutions review referrals and schedule multidisciplinary assessments [7].
4. Specialists diagnosing and treating the patient at ‘second-level’ institutions.
5. Reverse referrals occur when ‘second-level’ institutions discharge patients to ‘first-level’ institutions for rehabilitation, supported by detailed discharge summaries and follow-up plans: follow-up consultations *via* telemedicine will ensure continuity of care.

Additionally, cultivating ‘first-level’ institutions specialising in rare disease recovery and providing regular training for health care workers is crucial for maintaining quality standards [8]. Moreover, complementary strategies should be developed, including the exploration of specialised insurance payment mechanisms for rare diseases [9]. Examples include direct settlement systems that simplify reimbursement for cross-regional or cross-level care by reducing upfront payments and administrative burdens, as well as bundled payment models that cover the entire care journey to promote integrated and cost-effective care.

## BUILDING A DIGITAL PLATFORM: USING NOVEL TECHNOLOGIES

Leveraging artificial intelligence (AI), blockchain, and big data technologies can enable the comprehensive management of rare disease referrals through digital connectivity and collaboration. First, overcoming data barriers by establishing a unified, horizontally and vertically integrated collaborative network will make hospital capacity data transparent, patient referral processes accessible, and referral applications and approvals intelligent. Second, enhancing the rare disease screening and rehabilitation capabilities of ‘first-level’ medical institutions through telemedicine, intelligent training platforms, and digital follow-up monitoring can help reduce unnecessary patient transfers [10,11]. For instance, an AI-based smartphone application has been developed to screen for 128 rare genetic disorders using facial photographs, enabling early detection at primary care levels and reducing diagnostic delays [12]. Third, the ongoing development of AI technologies will improve the accuracy of rare disease diagnoses in ‘second-level’ institutions, streamline treatment processes, and reduce the occurrence of adverse events [13]. Lastly, a supervised flow of information is proposed, using blockchain technology to ensure data security and transparency [14]. Blockchain will manage data ownership by providing patients with control over their health information through private keys, which grant access only to authorised personnel. Access rights will be carefully managed via smart contracts, ensuring that data usage is logged and auditable to maintain compliance with privacy regulations. As demonstrated by the Swarm learning model, it allows for secure, decentralised sharing of patient data across institutions, ensuring privacy while fostering collaboration in rare disease diagnosis and treatment [15].

## INTEGRATING WITH EXISTING HEALTH CARE SYSTEM: OVERCOMING POTENTIAL CHALLENGES

Building upon the existing system, the proposed model would integrate specialised rare disease capabilities into the existing structure while also improving referral pathways, reducing inefficiencies, and creating synergistic effects within the health care infrastructure. The integration process will involve several steps. First, conducting a comprehensive assessment of current hospital capabilities to categorise them into first and secondary levels. Second, developing specialised training programmes to enhance the corresponding skills of health care providers at both levels. Third, implementing digital platforms for seamless referral, communication, and data sharing. Fourth, establishing a continuous feedback mechanism to monitor and refine the integration.

The proposed model is broadly applicable to health care systems around the world. Tailoring it to local health care infrastructure, funding, and workforce availability is critical to ensure its scalability and effectiveness globally.

Certainly, challenges exist during the process. These may include resistance to systemic change (*e.g.* health care providers may be concerned about increased workload or redistribution of resources), infrastructure limitations, and funding constraints [16]. Engaging stakeholders through workshops and pilot programmes, investing in infrastructure upgrades and telemedicine platforms, and seeking funding from public-private partnerships and government grants. Additionally, conducting pilot programmes in select regions is essential for observing real-world outcomes [17]. We thus propose a phased five-year timeline: programme design and setup for year one, model implementing and scaling up for years two and three, and full implementation and continuous monitoring for years four and five. Cost-benefit analyses could measure the effectiveness and practicality of the two-tier system on diagnostic timelines and health care costs.

## INCENTIVISING STAKEHOLDER ENGAGEMENT: DEVELOPING TRAINING AND ADVOCACY PLAN

To ensure the successful implementation and adoption of the proposed system, it is essential to develop a comprehensive training and advocacy plan for both patients and health care practitioners. For health care providers, regular training sessions should be conducted to enhance their understanding of rare diseases, improve their diagnostic and treatment capabilities, and familiarise them with the new two-tier hierarchical structure and digital referral network. This training could be delivered through interactive workshops, online modules, and telemedicine platforms, ensuring accessibility and flexibility [18]. Additionally, health care practitioners should be incentivised through performance-based evaluations, financial rewards, and professional development opportunities tied to their engagement with the system.

For patients, targeted education campaigns should be launched to raise awareness about the availability of rare disease services, the referral network, and the benefits of engaging with the new system. These campaigns could include multilingual informational materials, community outreach programmes, and digital tools such as mobile apps for navigation and appointment scheduling [19]. Advocacy groups and patient ambassadors should co-develop culturally relevant resources and share success stories to build trust and encourage participation [20]. Feedback mechanisms should also be integrated into the plan to assess its effectiveness. Periodic surveys and focus groups can help identify barriers to engagement, while pilot programmes in select regions can test and refine the strategies before scaling up nationwide [21].

## CONCLUSIONS

Transforming the hierarchical medical system is imperative to addressing the challenges faced by patients with rare diseases. By implementing a two-tier structure that classifies medical institutions based on their diagnostic and treatment capabilities, we can enhance the efficiency and effectiveness of care. Establishing an efficient one-stop referral network will streamline patient pathways, ensuring timely access to specialised services. Leveraging digital technologies will bridge information gaps, facilitate collaboration, and optimise resource utilisation. Together, these strategies will improve patient outcomes, reduce health care burdens, and contribute to the global goal of ensuring healthy lives and promoting well-being.
